# Estimation of gestational age in early pregnancy from crown-rump length when gestational age range is truncated: the case study of the INTERGROWTH-21^st^ Project

**DOI:** 10.1186/1471-2288-13-151

**Published:** 2013-12-07

**Authors:** Eric O Ohuma, Aris T Papageorghiou, Jose Villar, Douglas G Altman

**Affiliations:** 1 Nuffield Department of Obstetrics & Gynaecology and Oxford Maternal & Perinatal Health Institute (OMPHI), Green Templeton College, University of Oxford, Level 3 Women's Centre, John Radcliffe Hospital, Headington, Oxford OX3 9DU, UK; 2 Centre for Statistics in Medicine, University of Oxford, Botnar Research Centre, Windmill Road, Oxford OX3 7LD, UK

**Keywords:** Truncation, INTERGROWTH-21^st^ project, Crown-rump length, Gestational age, Simulation, Extrapolation, Restriction, Inversion

## Abstract

**Background:**

Fetal ultrasound scanning is considered vital for routine antenatal care with first trimester scans recommended for accurate estimation of gestational age (GA). A reliable estimate of gestational age is key information underpinning clinical care and allows estimation of expected date of delivery. Fetal crown-rump length (CRL) is recommended over last menstrual period for estimating GA when measured in early pregnancy i.e. 9^+0^-13^+6^ weeks.

**Methods:**

The INTERGROWTH-21^st^ Project is the largest prospective study to collect data on CRL in geographically diverse populations and with a high level of quality control measures in place. We aim to develop a new gestational age estimation equation based on the crown-rump length (CRL) from women recruited between 9^+0^-13^+6^ weeks. The main statistical challenge is modelling data when the outcome variable (GA) is truncated at both ends, i.e. at 9 and 14 weeks.

We explored three alternative statistical approaches to overcome the truncation of GA. To evaluate these strategies we generated a data set with no truncation of GA that was similar to the INTERGROWTH-21^st^ Project CRL data, which we used to explore the performance of different methods of analysis of these data when we imposed truncation at 9 and 14 weeks of gestation. These 3 methods were first tested in a simulation based study using a previously published dating equation by Verburg et al. and evaluated how well each of them performed in relation to the model from which the data were generated. After evaluating the 3 approaches using simulated data based on the Verburg equations, the best approach will be applied to the INTERGROWTH-21^st^ Project data to estimate GA from CRL.

**Results:**

Results of these rather “ad hoc” statistical methods correspond very closely to the “real data” for Verburg, a data set that is similar to the INTERGROWTH-21^st^ project CRL data set.

**Conclusions:**

We are confident that we can use these approaches to get reliable estimates based on INTERGROWTH-21^st^ Project CRL data. These approaches may be a solution to other truncation problems involving similar data though their application to other settings would need to be evaluated.

## Background

Fetal ultrasound scanning is considered an essential part of routine antenatal care with first trimester scans recommended for confirming viability, accurate estimation of gestational age and determining the number of fetuses [1,2]. Fetal crown-rump length (CRL) is measured in early pregnancy primarily to determine the gestation age (GA) of a fetus and is most reliable between 9^+0^ to 13^+6^ weeks’ gestation, but not beyond [3]. Assessment of gestational age based on ultrasound (US) biometry was first introduced in 1969 by Campbell [4], and it has become the preferred method for dating pregnancy.

A reliable estimate of gestational age is key information as it underpins clinical care and allows estimation of the expected date of delivery. There are 3 ways to estimate gestational age early in pregnancy: a) based on a reliable first day of the last menstrual period (LMP) alone; b) based on an early (9^+0^ to 13^+6^ weeks) ultrasound alone, or c) LMP and ultrasound combined. Use of LMP is based on the assumption that pregnancy has a constant duration from the first day of the LMP with ovulation on the 14^th^ day [3]. This method of dating pregnancies, even for women whose menstrual history is certain, has been shown to be unreliable [5,6]. Caution is recommended regarding use of last menstrual period (LMP) alone for dating because up to 50% of women are uncertain of their dates, have an irregular cycle, have recently stopped the oral contraceptive pill, are lactating or did not have a normal last menstrual period [7].

The National Institute for Health and Care Excellence (NICE) Guideline for Routine Antenatal Care (2008) and International Society of Ultrasound in Obstetrics and Gynaecology (ISUOG) recommend that all pregnant women should be offered an early US examination to date pregnancies [1,7,8]. It is stated that ideally this should be performed by the measurement of CRL between 10 and 13^+6^ weeks which can reduce the need for induction of labour after 41 weeks of gestation. Although there is always a margin of error in US-based estimation [9], this error is relatively small compared to LMP-based estimations [8,10].

Many dating charts are now in use though developed from different populations resulting in discrepancies when compared or applied to a specified population hence there is a need for an international reference dating equation and chart [11-15]. The INTERGROWTH-21^st^ Project, described below, aims to generate fetal growth charts and also a new dating chart. In the study gestational age is based on the first day of LMP and corroborated by CRL using a known dating equation [16]. Therefore, only women between 9^+0^-13^+6^ weeks gestation whose estimation by both methods agreed within 7 days were recruited into the fetal growth longitudinal study.

To develop charts of fetal size we need to model CRL as a function of GA while for dating we interchange the variables and model GA as a function of CRL. This latter analysis is problematic if the available data are constrained by a restricted range of GA [17]; such a restriction is commonly in place, as fetal curling prevents accurate measurement beyond 13^+6^ weeks. In this paper we describe an exploration of strategies to overcome truncation of GA when developing equations and charts for dating pregnancies from CRL measurements.

## Methods

The International Fetal and Newborn Growth Consortium for the 21^st^ Century (INTERGROWTH-21^st^) is a large-scale, population-based, multi-centre project involving health institutions from eight geographically diverse countries (i.e. Brazil, China, India, Oman, Kenya, UK, USA and Italy), which aims to assess fetal, newborn and preterm growth under optimal conditions, in a manner similar to that adopted by the WHO Multicentre Growth Reference Study [18]. This approach is important in the creation of fetal growth standards by selecting women regarded as “healthy”, educated, affluent and living in areas with minimal environmental constraints on growth [19].

The INTERGROWTH-21^st^ Project has three major components, which were designed to create: 1) Longitudinally derived, prescriptive, international, fetal growth standards using both clinical and ultrasound measures; 2) Preterm, postnatal growth standards for those infants born ≥26^+0^ but <37^+0^ weeks of gestation in the longitudinal cohort, and 3) Birth weight, newborn length, and head circumference for gestational age standards derived from all newborns delivering at the study sites over an approximately 12 month period [19]. To ensure that ultrasound measurements are accurate and reproducible, centres adopted uniform methods, used identical ultrasound equipment in all the study sites; adopted standardised methodology to take fetal measurements, and employed locally accredited ultra-sonographers who underwent standardisation training and monitoring.

One aim of the longitudinal study of the INTERGROWTH-21^st^ Project is to develop a new gestational age estimation equation based on the crown-rump length (CRL) from women recruited between 9^+0^-13^+6^ weeks. This will be the largest prospective study to collect data on CRL in geographically diverse populations, and with a high level of quality control measures in place.

Several reliable statistical methods exist for developing age-related reference centiles [20-22]. These can be applied in a straightforward way for developing equations for fetal size as function of GA. For dating, however, we need to estimate GA as a function of fetal size, specifically the fetal CRL. We sought to use the INTERGROWTH-21^st^ data to develop centiles for the distribution of GA for CRL values between 15 mm and 100 mm. The statistical challenge is this: How can we model data when the outcome variable (GA) is truncated at both ends, i.e. at 9 and 14 weeks, given the need to obtain estimates in the truncated regions? This restriction is part of the design of the INTERGROWTH-21^st^ study based on the fact that CRL measurements are less reliable outside this range of GA [1,7,23-25].

Ignoring the truncation of GA would lead to seriously biased estimates. We explored three alternative statistical approaches to overcome the truncation of GA. To evaluate these strategies we generated a data set with no truncation of GA that was similar to the INTERGROWTH-21^st^ Project CRL data, which we used to explore the performance of different methods of analysis of these data when we imposed truncation at 9 and 14 weeks of gestation. The choice of which approach is best is hard to justify through formal statistical testing, and is likely to depend on the specific data being analysed.

### Statistical methods

Data were explored visually by a scatter plot of CRL by GA and vice versa. The relationship between GA and CRL is non-linear though the distribution of CRL is conditionally normal at any given gestational age. By contrast GA has a positively skewed distribution for a given CRL [17]. We applied fractional polynomial (FP) models (which are very flexible) to the data by fitting separate models to the mean and standard deviation (SD) of GA to account for increase in variance with greater CRL and gestation [20,22]. Using equations of the mean and standard deviation one can easily compute any desired centiles using the relation

$$P^{\mathrm{th}}\text{centile}=\text{Median}\;\text{CRL}+K\text{SD}$$

where *K* is the normal equivalent deviate (z score) corresponding to a particular centile, e.g. *K* = 1.88 for the 97^th^ centile and -1.88 for the 3^rd^ centile, and the SD in this equation are the predicted estimates from the regression analysis. Fitted curves (3^rd^, 50^th^, and 97^th^ centiles) from different models were assessed visually for a good fit and by comparing the deviances from each model. The choice of centiles presented was purely based on what is commonly reported in the literature and also used in clinical practice as standard centiles. In addition; the INTERGROWTH-21^st^ Project aims to complement the WHO-Multi-centre Growth Reference Study (MGRS) which produced reference standards for children aged 0-5 years where they also presented the 3^rd^ and 97^th^ centiles [18]. Goodness of fit was assessed by a scatter plot of the distribution of residuals in z scores by CRL and also by counting the number of observations below the 3^rd^ and above the 97^th^ centiles.

We explored three approaches to deal with truncation of gestational age at 9 and 14 weeks by (a) Simulation, Restriction and Extrapolation (b) Simulation (c) Inversion of model for predicting CRL from GA. Extrapolation was applied purely for the purposes of obtaining reliable estimates between 9 and 14 weeks in the presence of truncation at 9 weeks and 14 weeks. The resultant equation will not be used for dating beyond 14 weeks as this is not recommended in clinical practice. The reliability of fractional polynomial models for extrapolation has been discussed previously by Royston & Altman where they show that fractional polynomial models extrapolate well at least for fetal measurements [22]. These 3 methods were first tested in a simulation based study using a previously published dating equation by Verburg et al. [2]. We evaluated how well each of the 3 approaches performed in relation to the model from which the data were generated.

The Verburg equation was selected from the many dating equations in use as it is one of the five preferred dating equations according to a recent systematic review of the methodology used for creating dating charts [13]; it is also recommended by the International Society of Ultrasound in Obstetrics and Gynaecology (ISUOG) [1,13]. The great strength of performing a simulation study based on a known dating equation is that it allows us to evaluate how well our proposed methods of dealing with truncation perform in a situation where we know the “truth” (i.e. the equations from which simulated data were obtained). After evaluating the 3 approaches using simulated data based on the Verburg equations, the best approach will be applied to the INTERGROWTH-21^st^ Project data to estimate GA from CRL.

Data were simulated from Verburg’s dating equations [2]:

$$\begin{array}{l} \text{Mean}\;\mathrm{of}\;\text{log}\;\text{GA}=1.4653+0.001737\times \mathrm{CRL}\\ \;+0.2313\times \text{log}\;\text{CRL} \end{array}$$

SDoflogGA=0.04590

Here and throughout all logarithms are natural logarithms.

These equations assume that log GA has a normal distribution for any value of CRL. From these equations we simulated 100 observations for each CRL value from 5 mm and 110 mm in 1 mm increments, resulting in 10,600 observations in total. A sample size of 100 was chosen as it represented the average number of CRL observations for each GA in the INTERGROWTH-21^st^ data and is large enough to remove effects of sampling variation. The GA was between 5 and 17 weeks, the GA range of original data from which the equations were obtained. We log transformed GA in all analyses to stabilise variance [2,15,20,26].

### Validation of the simulated data

We modelled the simulated data using fractional polynomial regression of log transformed GA on CRL and compared the fractional polynomial (FP) terms and the predicted median GA from the equation obtained to the original dating equation reported by Verburg et al. The equations obtained from simulated data were remarkably similar to Verburg’s original equations:

$$\begin{array}{l} \text{Mean}\;\mathrm{of}\;\text{log}\;\text{GA}=1.4612+0.001693\times \text{CRL}\\ \;+0.2332\times \text{log}\;\text{CRL} \end{array}$$

SDoflogGA=0.0458114-0.00000198×CRL

**Figure 1:**
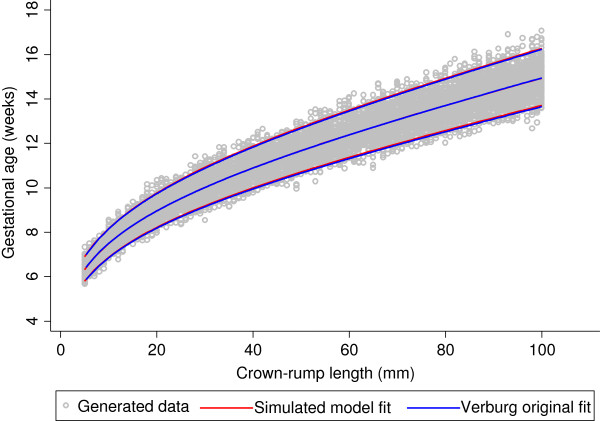
**Simulated data for crown-rump length measurements in relation to gestational age with fitted centiles.** Full title: Simulated data for crown-rump length (CRL) measurements in relation to gestational age (grey circles) with 3^rd^ and 97^th^ fitted centiles. Blue continuous lines represent the original equation fit reported by Verburg et al. [2] and from which the simulated data are derived whereas the red continuous lines represent model fit of the simulated data.

Both equations for the median were FP models of degree 2 with powers 0 and 1 (i.e. terms in CRL and log CRL). The equation for SD was a FP model of degree 1, power 1 (linear), compared to the SD obtained by Verburg which was a constant. The predicted GA from the two equations agreed within 0.08 days (Figure 1, Table 1).

**Table 1:** **Crown-rump length (CRL) measurements in relation to gestational age for the original equation fit reported by Verburg et al.**[2]**compared to our model fit of the simulated data**

	**Verburg’s original reported equation**	**Equation from the simulated data**	
**CRL (mm)**	**Median GA (Weeks) predicted from CRL**	**Median GA (Weeks) predicted from CRL**	**Difference in GA (days)**
5	6.336	6.324	0.082
10	7.503	7.497	0.041
15	8.312	8.310	0.015
20	8.962	8.962	-0.003
25	9.519	9.521	-0.017
30	10.015	10.019	-0.026
35	10.469	10.474	-0.032
40	10.892	10.897	-0.035
45	11.290	11.296	-0.036
50	11.670	11.675	-0.036
55	12.034	12.039	-0.033
60	12.386	12.390	-0.029
65	12.727	12.731	-0.023
70	13.060	13.063	-0.016
75	13.386	13.387	-0.008
80	13.706	13.706	0.001
85	14.021	14.019	0.012
90	14.331	14.328	0.023
95	14.638	14.633	0.036
100	14.942	14.935	0.050

**Figure 2:**
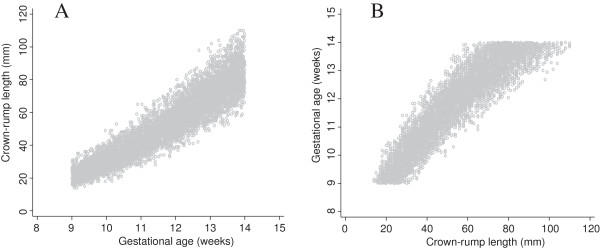
**Simulated data generated from dating equation by Verburg and truncated at 9 and 14 weeks.** Full title: Simulated data generated from the dating equation by Verburg et al. and truncated at 9 and 14 weeks. Panel **A** shows crown-rump length (CRL) versus gestational age for creating a size chart and panel **B** shows gestational age versus crown-rump length for creating a dating chart.

After successful validation of the simulated data we truncated gestational age at 9 and 14 weeks to match the INTERGROWTH 21^st^ data set. We note that truncation is only a problem when we want to model GA as a function of CRL and not CRL as a function of GA (size chart) (Figure 2, panel A). All three suggested approaches make use of this fact, but in different ways.

**Figure 3:**
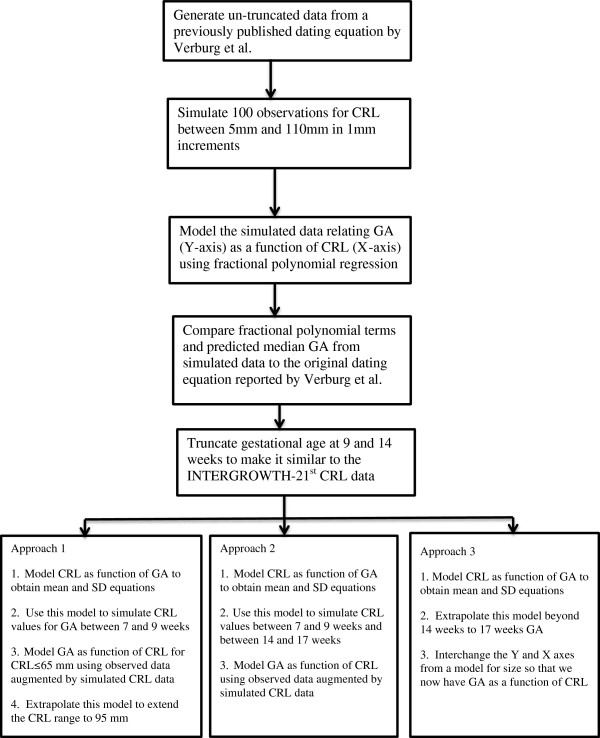
**A Flow diagram summarising the process and methodology of the simulation study.** Full title: A Flow diagram summarising the process and methodology of the simulation study to evaluate three methods to overcome the truncation problem inherent in the data set.

We applied the three proposed approaches to the truncated simulated data shown in Figure 2. Figure 3 shows a flow diagram summarising all the three methods.

### Approach 1-simulation for small crown-rump length, restriction and extrapolation

**Figure 4:**
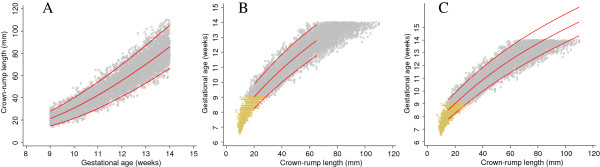
**Crown-rump length measurements in relation to gestational age with fitted centiles (Approach 1).** Full title: Crown-rump length (CRL) measurements in relation to gestational age (grey circles) with 3^rd^, 50^th^ and 97^th^ fitted centiles (Panel **A**). Yellow small crosses in panels **B** and **C** represent data simulated from the fitted equation of the mean and SD from panel **A**. Panel **B** shows the model fit relating GA and CRL with CRL restricted to ≤ 65 mm and panel **C** shows the model fit in panel **B** extrapolated to the full range of CRL (Approach 1).

The first approach is based on first modelling CRL as a function of GA (Figure 4, panel A). From the obtained equation of the median GA, we simulate 100 CRL observations (about the same number of observations for each day of GA in the un-truncated data set) for each day of gestation between 7 and 9 weeks, to overcome the truncation at the bottom end of the distribution of CRL measurements. The choice of 7 weeks as a lower limit for extrapolation was based on the desire to be able to obtain a good fit to the data at 9 weeks where the actual data is truncated and it was also the lowest limit where the fitted equations and range of gestational age remained plausible when extrapolated. Then, using the augmented data set, we model GA as a function of CRL with CRL restricted to ≤ 65 mm (lowest CRL measurement reported at 14 weeks in the INTERGROWTH-21^st^ data set) as there remains a truncation problem at the upper end of the CRL distribution (Figure 4, panel B). We then extrapolated the mean and SD equations obtained to the rest of the data (Figure 4, panel C). The predicted GA from this approach was compared to that originally reported by Verburg (Table 2). A sensitivity analysis to establish which lower cut-off, i.e. truncating CRL at 10 mm, 15 mm or 20 mm had the best prediction, was performed by comparing the predicted GA obtained using the derived equation to that reported by Verburg. We note that the choice of a cut-off affects the fit for large CRL and so has clinical implications, because it is desirable to have predictions of GA from CRL between 15 mm and 95 mm (Table 2).

**Table 2:** Estimated gestational age in relation to crown-rump length (CRL) measurements for the original equation reported by Verburg and a model fitted to the simulated data (Approach 1)

	**Verburg’s original equation**			**Approach 1**					
	**Estimated GA (weeks)**			**Estimated GA (weeks)**			**Difference (days)**		
**CRL (mm)**	**3**^**rd **^**centile**	**Median**	**97**^**th **^**centile**	**3**^**rd **^**centile**	**Median**	**97**^**th **^**centile**	**3**^**rd **^**centile**	**Median**	**97**^**th **^**centile**
10	6.88	7.50	8.18	6.85	8.18	8.22	0.21	-4.76	-0.28
15	7.63	8.31	9.06	7.60	8.53	9.09	0.21	-1.54	-0.21
20	8.22	8.96	9.77	8.20	9.02	9.80	0.14	-0.42	-0.21
25	8.73	9.52	10.38	8.72	9.51	10.40	0.07	0.07	-0.14
30	9.19	10.02	10.92	9.18	9.99	10.93	0.07	0.21	-0.07
35	9.60	10.47	11.41	9.60	10.45	11.41	0.00	0.14	0.00
40	9.99	10.89	11.87	10.00	10.88	11.86	-0.07	0.07	0.07
45	10.36	11.29	12.31	10.37	11.30	12.29	-0.07	-0.07	0.14
50	10.70	11.67	12.72	10.73	11.69	12.69	-0.21	-0.14	0.21
55	11.04	12.03	13.12	11.08	12.07	13.07	-0.28	-0.28	0.35
60	11.36	12.39	13.50	11.41	12.43	13.44	-0.35	-0.28	0.42
65	11.67	12.73	13.87	11.74	12.77	13.80	-0.49	-0.28	0.49
70	11.98	13.06	14.24	12.05	13.11	14.15	-0.49	-0.35	0.63
75	12.28	13.39	14.59	12.37	13.43	14.49	-0.63	-0.28	0.70
80	12.57	13.71	14.94	12.67	13.74	14.82	-0.70	-0.21	0.84
85	12.86	14.02	15.28	12.98	14.04	15.15	-0.84	-0.14	0.91
90	13.15	14.33	15.62	13.27	14.34	15.47	-0.84	-0.07	1.05
95	13.43	14.64	15.96	13.57	14.62	15.79	-0.98	0.14	1.19
100	13.71	14.94	16.29	13.86	14.90	16.10	-1.05	0.28	1.33

### Approach 2 – simulation for small and large crown-rump length

**Figure 5:**
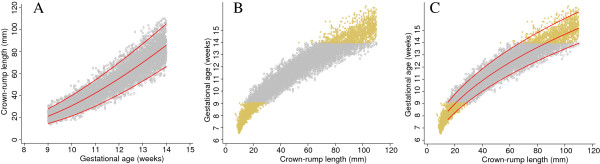
**Crown-rump length measurements in relation to gestational age with fitted centiles (Approach 2).** Full title: Crown-rump length (CRL) measurements in relation to gestational age (grey circles) with 3^rd^, 50^th^, and 97^th^ fitted centiles (Panel **A**). Yellow small crosses in panels **B** and **C** represent data simulated from the fitted equation of the mean and SD from panel **A**. Panel **C** shows the model fit relating GA and CRL (Approach 2).

Approach 2 is very similar to Approach 1, with data simulated from fitting a size equation and using the mean and SD equations of CRL by log GA (Figure 5, panel A). We use the model for CRL to simulate 100 observations of CRL (about the same number of observations for each day of GA in the un-truncated data set) for each day of gestation at both ends of the distribution, i.e. below 9 weeks (between 7 and 9 weeks) and above 14 weeks (between 14 and 17 weeks) of gestation (Figure 5, panel B). The choice of 7 weeks as a lower limit and 17 weeks as an upper limit for extrapolation was based on the desire to be able to obtain a good fit to the data between 9 and 14 weeks where the actual data is truncated. The two cut-offs (at 7 and 17 weeks) were also the lowest and upper limits where the fitted equations and range of gestational age remained plausible when extrapolated.

The simulated CRL measurements below 9 weeks and above 14 weeks overcomes the truncation problem presented by the data thereby allowing us to model GA as a function of CRL more efficiently and obtain the respective median and SD equation (Figure 5, panel C). The predicted GA from this approach was compared to that originally reported by Verburg (Table 3). A sensitivity analysis assessment was performed in relation to the value of the lower end cut-off of CRL.

**Table 3:** Crown-rump length (CRL) measurements in relation to gestational age for the original equation fit reported by Verburg compared to model fit of the simulated data (Approach 2)

	**Verburg’s original equation**			**Approach 2**					
	**Estimated GA (weeks)**			**Estimated GA (weeks)**			**Difference (days)**		
**CRL (mm)**	**3**^**rd **^**centile**	**Median**	**97**^**th **^**centile**	**3**^**rd **^**centile**	**Median**	**97**^**th **^**centiles**	**3**^**rd **^**centile**	**Median**	**97**^**th **^**centile**
10	6.88	7.50	8.18	7.08	7.71	8.39	-1.41	-1.45	-1.48
15	7.63	8.31	9.06	7.70	8.38	9.12	-0.52	-0.47	-0.41
20	8.22	8.96	9.77	8.25	8.98	9.77	-0.21	-0.12	-0.02
25	8.73	9.52	10.38	8.75	9.52	10.36	-0.11	0.00	0.12
30	9.19	10.02	10.92	9.20	10.01	10.90	-0.09	0.02	0.15
35	9.60	10.47	11.41	9.62	10.47	11.39	-0.11	0.01	0.14
40	9.99	10.89	11.87	10.01	10.89	11.86	-0.13	-0.01	0.13
45	10.36	11.29	12.31	10.38	11.29	12.29	-0.14	-0.02	0.12
50	10.70	11.67	12.72	10.72	11.67	12.70	-0.13	0.00	0.15
55	11.04	12.03	13.12	11.05	12.03	13.09	-0.09	0.04	0.20
60	11.36	12.39	13.50	11.37	12.37	13.46	-0.03	0.11	0.28
65	11.67	12.73	13.87	11.67	12.70	13.82	0.06	0.21	0.39
70	11.98	13.06	14.24	11.96	13.01	14.16	0.18	0.35	0.54
75	12.28	13.39	14.59	12.23	13.31	14.49	0.33	0.51	0.73
80	12.57	13.71	14.94	12.50	13.60	14.81	0.50	0.71	0.95
85	12.86	14.02	15.28	12.76	13.89	15.11	0.71	0.94	1.20
90	13.15	14.33	15.62	13.01	14.16	15.41	0.95	1.20	1.49
95	13.43	14.64	15.96	13.25	14.42	15.70	1.22	1.50	1.81
100	13.71	14.94	16.29	13.49	14.68	15.98	1.51	1.82	2.17

### Approach 3 – interchanging the X and Y axes from a model for size

**Figure 6:**
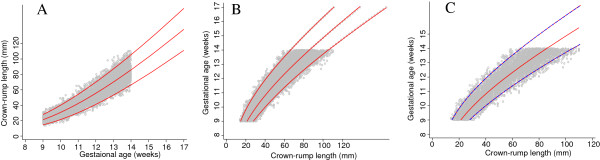
**Crown-rump length measurements in relation to gestational age with fitted centiles (Approach 3).** Full title: Crown-rump length (CRL) measurements in relation to gestational age (GA) with fitted 3^rd^, 50^th^ and 97^th^ centiles (Panel **A**). Panel **B** shows the relation between GA and CRL after interchanging the axes and fitting new models to the three sets of coordinates. Panel **C** shows the model obtained by simply taking the average of 2 SDs. An equation for the SD relating GA to CRL was then obtained by regressing this SD (of GA) on CRL and estimating outer centiles by combining the model for SD with that for the median (Approach 3).

The third approach does not require simulating data. As before, we model CRL (Y axis) as a function of GA (X axis) using all the available data. We then extrapolate the obtained equations to larger GA to cover the desired range of CRL (Figure 6, panel A). We then interchange the X and Y axes to give GA (Y-axis) as a function of CRL (X-axis) (Figure 6, panel B). We do not now have equations for the median and SD describing the relationship between GA to CRL but rather three sets of X, Y coordinates of GA giving the predicted 3^rd^, 50^th^ and 97^th^ centiles for CRL. We can obtain a new equation for the median by regressing GA on the predicted median CRL. Similarly, we can obtain equations for the 3^rd^ and 97^th^ centiles (Figure 6, panel C). The predicted GA from this approach was compared to that originally reported by Verburg (Table 4). Since we do not have an equation for the SD, the full model cannot be written down simply. We describe how we obtained an equation for the SD as function of CRL that also allows prediction of any desired centiles.

**Table 4:** Crown-rump length (CRL) measurements in relation to gestational age for the original equation fit reported by Verburg compared to model fit of the simulated data (Approach 3)

	**Verburg’s original equation**			**Approach 3**					
	**Estimated GA (weeks)**			**Estimated GA (weeks)**			**Difference (days)**		
**CRL (mm)**	**3**^**rd **^**centile**	**Median**	**97**^**th **^**centile**	**3**^**rd **^**centile**	**Median**	**97**^**th **^**centiles**	**3**^**rd **^**centile**	**Median**	**97**^**th **^**centile**
10	6.88	7.50	8.18	6.97	7.29	8.15	-0.60	1.49	0.23
15	7.63	8.31	9.06	7.66	8.17	9.08	-0.26	1.00	-0.14
20	8.22	8.96	9.77	8.23	8.85	9.81	-0.10	0.79	-0.25
25	8.73	9.52	10.38	8.73	9.42	10.42	-0.02	0.68	-0.29
30	9.19	10.02	10.92	9.19	9.93	10.96	0.00	0.62	-0.29
35	9.60	10.47	11.41	9.60	10.39	11.45	0.00	0.57	-0.28
40	9.99	10.89	11.87	9.99	10.81	11.91	-0.02	0.54	-0.26
45	10.36	11.29	12.31	10.36	11.22	12.34	-0.05	0.52	-0.24
50	10.70	11.67	12.72	10.72	11.60	12.75	-0.08	0.50	-0.22
55	11.04	12.03	13.12	11.05	11.96	13.15	-0.11	0.48	-0.20
60	11.36	12.39	13.50	11.38	12.32	13.53	-0.14	0.47	-0.18
65	11.67	12.73	13.87	11.70	12.66	13.90	-0.16	0.47	-0.15
70	11.98	13.06	14.24	12.01	12.99	14.25	-0.17	0.47	-0.12
75	12.28	13.39	14.59	12.30	13.32	14.61	-0.18	0.47	-0.09
80	12.57	13.71	14.94	12.60	13.64	14.95	-0.18	0.48	-0.05
85	12.86	14.02	15.28	12.88	13.95	15.29	-0.16	0.49	-0.01
90	13.15	14.33	15.62	13.17	14.26	15.62	-0.14	0.52	0.04
95	13.43	14.64	15.96	13.44	14.56	15.94	-0.11	0.55	0.10
100	13.71	14.94	16.29	13.72	14.86	16.27	-0.06	0.58	0.16

### Computing an equation for the standard deviation

We have described above how to obtain equations for say the 3^rd^, 50^th^ and 97^th^ centiles by regressing GA on the predicted *p*^th^ centile of CRL measurements. Using these equations (3^rd^, 50^th^ and 97^th^ centile) relating log GA and CRL we can get two estimates of the SD at a given CRL from the difference between 97^th^ and 50^th^ centiles and between the 50^th^ and 3^rd^ centiles. Note that the two are not exactly the same but are very similar because GA was modelled on the log scale. It is thus reasonable to estimate the SD for each value of CRL by simply taking the average of the 2 SDs. An equation for the SD relating GA to CRL was then obtained by regressing this SD (of GA) on CRL. Estimates of any desired centiles can then be obtained using the relation:

$$P^{\text{th}}\text{centile}=\text{Median}\;\text{CRL}+K\text{SD}$$

where *K* is the normal equivalent deviate (z score) corresponding to a particular centile, e.g. *K* = 1.88 for the 97^th^ centile and -1.88 for the 3^rd^ centile, and the SD in this equation are the predicted estimates from the regression analysis just described.

## Results

**Figure 7:**
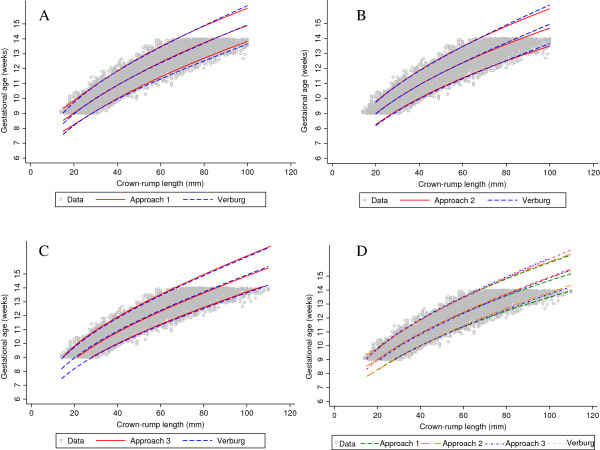
**Crown-rump length measurements in relation to gestational age comparing the 3 approaches with Verburg.** Full title: Crown-rump length (CRL) measurements in relation to gestational age for the simulated data for CRL from 9^+0^ to 13^+6^ weeks gestational age comparing each of the 3 approaches with Verburg (Panel **A**, **B** and **C**) and all the 3 approaches with Verburg (Panel **D**).

The agreement in estimated median GA between approach 1 and Verburg’s original fit was within 0.4 days for CRL between 20 mm and 100 mm. The largest difference was at the lower range of CRL i.e. 4.8 days and 1.5 days for CRL values of 10 mm and 15 mm respectively (Figure 4, Table 2, and Figure 7). This is notably because the model was first fit for CRL between 20 mm and 65 mm and extrapolated to the rest of the data. Model fits beginning with lower CRL values i.e. 10 mm and 15 mm did not perform as well when extended to the rest of the data. There were 135/4600 (2.9%) observations below the 3^rd^ centile and 120/4600 (2.6%) above the 97^th^ centile for CRL between 20 mm and 100 mm (Figure 4).

The predicted values of median GA from approach 2 agreed within 1 day for CRL between 15 mm and 85 mm with the largest difference at the 2 extremes of CRL, i.e. 1.5 days for CRL of 10 mm and 1.8 days for CRL of 100 mm (Figure 5, Table 3, and Figure 7). There were 207/7640 (2.7%) observations below the 3^rd^ centile and 232/7640 (3.0%) above the 97^th^ centile for CRL between 20 mm and 100 mm (Figure 5).

Approach 3 agreed within 1 day for CRL between 15 mm and 100 mm with the largest difference of 1.5 days observed at CRL of 10 mm. Approach 3 underestimated the predicted median GA across the whole range by ~0.6 days (Figure 6, Table 4, and Figure 7). There were 128/6448 (2.0%) observations below the 3^rd^ centile and 221/6448 (3.4%) above the 97^th^ centile for CRL between 20 mm and 100 mm (Figure 6). The estimates obtained from the computation of SD for approach 3 were remarkably similar to those obtained from the three sets of X, Y coordinates of GA and the predicted 3^rd^, 50^th^ and 97^th^ centiles for CRL (Figure 6 panels B and C).

**Figure 8:**
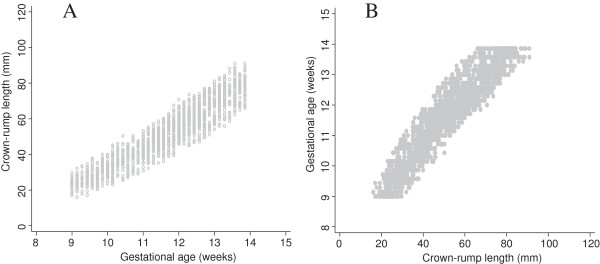
**Crown-rump length versus gestational age using a sample of the INTERGROWTH-21**^**st **^**CRL project data.** Full title: Crown-rump length (CRL) versus gestational age for creating a size chart (Panel **A**) and gestational age versus crown-rump length data for creating a dating chart (Panel **B**) using a sample of the INTERGROWTH-21^st^ project data (~35% of the overall target sample) for CRL from 9^+0^ to 13^+6^ weeks gestational age.

**Figure 9:**
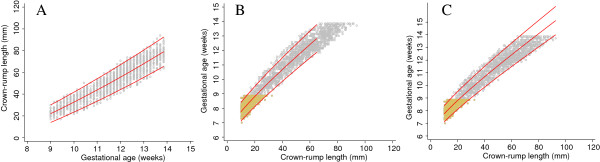
**INTERGROWTH-21**^**st **^**crown-rump length measurements in relation to gestational age with fitted centiles (Approach 1).** Full title: Crown-rump length (CRL) measurements in relation to gestational age (grey small hollow circles) with 3^rd^, 50^th^ and 97^th^ fitted centiles (Panel **A**). Brown small crosses in panel **B** and **C** represents the INTERGROWTH-21^st^ project data for CRL from 9^+0^ to 13^+6^ weeks gestational age of the fitted equation of the mean and SD from panel **A**. Panel **B** shows the model fit relating GA and CRL with CRL restricted to ≤ 65 mm and panel **C** shows the extrapolated model fit in panel **B** to the rest of the data (Approach 1).

**Figure 10:**
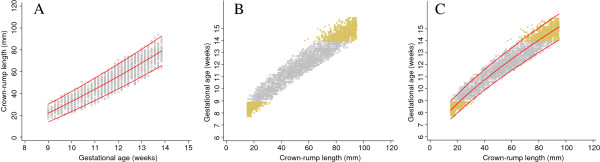
**INTERGROWTH-21**^**st **^**crown-rump length measurements in relation to gestational age with fitted centiles (Approach 2).** Full title: Crown-rump length (CRL) measurements in relation to gestational age (grey small hollow circles) with 3^rd^, 50^th^ and 97^th^ fitted centiles (Panel **A**). Brown small crosses in panels **B** and **C** represents the INTERGROWTH-21^st^ project data for CRL from 9^+0^ to 13^+6^ weeks gestational age of the fitted equation of the mean and SD from panel **A**. Panel **C** shows the model fit relating GA and CRL (Approach 2).

**Figure 11:**
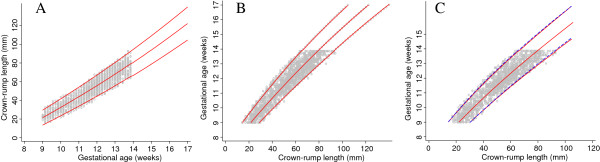
**INTERGROWTH-21**^**st **^**crown-rump length measurements in relation to gestational age with fitted centiles (Approach 3).** Full title: Crown-rump length (CRL) measurements in relation to gestational age (GA) (grey small hollow circles) with 3^rd^, 50^th^ and 97^th^ fitted centiles (Panel **A**). Panel **B** and **C** represents shows the relation between GA and CRL after interchanging the axes and refitting the model (Approach 3).

**Figure 12:**
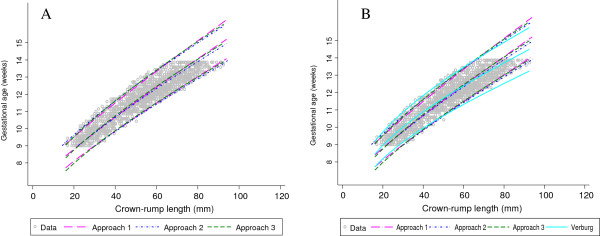
**INTERGROWTH-21**^**st **^**crown-rump length measurements in relation to gestational age comparing the 3 approaches with Verburg.** Full title: Crown-rump length (CRL) measurements in relation to gestational age for the INTERGROWTH-21^st^ project data for CRL from 9^+0^ to 13^+6^ weeks gestational age comparing all the 3 approaches (Panel **A**) and compared with Verburg (Panel **B**).

We have shown that these rather “ad hoc” approaches correspond very closely to the “real data” for Verburg (Figure 7), which is a data set that has similarities to the INTERGROWTH-21^st^ project CRL data set (Figure 8). Hence we are confident that we can use these approaches to get reliable estimates based on INTERGROWTH-21^st^ CRL data as demonstrated in the next section (Figures 9, 10, 11 and 12). We do not discuss any results of the INTERGROWTH-21^st^ CRL data as the data collection is still on-going and for demonstration purposes we have used ~35% of the overall target sample in this paper. Results of the full sample and the new international dating equation will be published in a separate paper.

Figure 8 shows data from 1600 fetuses (~35% of the overall target sample) included in the INTERGROWTH 21^st^ study, in the same format as Figure 2. The close similarity between the two data sets is apparent. The collection of INTERGROWTH-21^st^ data will be completed in 2013.

## Discussion

The main aim of this study was to explore the best methodology for modelling data when the outcome variable (GA) is truncated at both ends, i.e. at 9 and 14 weeks. We evaluated 3 approaches to overcome this difficulty by generating data from an existing equation (Verburg). The three approaches provided a good fit to the data (Figure 6) when compared to the original equation reported by Verburg. We appreciate that the choice of which approach is the best is hard to justify through formal statistical testing. Approach 2 was considered the best since it gives excellent results (i.e. estimates agreed within 1 day for CRL between 15 mm and 85 mm with the largest difference of 1.8 days at the very extreme end) when compared to approach 1 which had the largest difference (4.7 days) at the lower end of CRL distribution while approach 3 consistently underestimated GA by about half a day over the entire range of CRL.

A recent systematic review of CRL dating equations and charts showed large variations between studies with only very few studies reporting complete information on inclusion/exclusion criteria, maternal demographics, ultrasound quality control, last menstruation reliability and sample selection [13]. This potential for bias, methodological heterogeneity and limitations would affect clinical decision-making depending on the equation used; hence the need for an international dating equation and chart. The INTERGROWTH-21^st^ population which is carefully selected and actively followed up during pregnancy with a known outcome at birth provides a population that is ideal for developing such an international standard equation and chart. The INTERGROWTH-21^st^ project is the biggest study so far to prospectively collect data on CRL. These data are of very high quality, with ultrasound measurements made by highly trained sonographers following a standardised protocol using standard ultrasonography equipment with latest technology across 8 geographically diverse sites.

Gestational age estimation is an important component of clinical care and epidemiological studies. We believe that, as in other fields of medicine, all available information should be used for assessment, i.e. both LMP and ultrasound should be taken into account and agreement between the two required to be certain of its validity. One should consider that discrepancy between LMP and ultrasound could be due to disturbances in early fetal growth rather than an automatic assumption of incorrect dates, leading to re-dating. There is wide agreement that CRL is the best measure for assessing gestational age, certainly up to 14 weeks GA, since LMP is affected by both random error and systematic tendency to overstate the duration of gestation, biological variability and errors of the method including recall bias, digit preference, and additional bleeding after conception [5,27-32]. Ultrasound-based methods measure fetal size and use reliable LMP-based formulas (of which many are in use) to estimate gestational age; however this assumes no biological variability as all fetuses of a given size are estimated to have the same gestational age. However, biological variability exists and this is compounded by variability due to measurement error due to equipment and observer. Thus, accurate measurements of CRL require rigorous standardisation before initiation of the study and continuous quality control measures should be implemented similar to those routinely used in laboratory practices.

The implications of these different methods on research findings have recently been discussed [12]. Ultrasound can accurately determine the day of conception to within 5 days either way for 95% of cases and may be closer than LMP by an average of 2-3 days in predicting the date of a spontaneous delivery [1,17,27,28,33,34].

The unusual problem of truncation that we encountered in the INTERGROWTH-21^st^ CRL data is not unique in that it has been present in other studies, but has never been adequately addressed. This feature of the data has the potential to introduce considerable bias, mostly at the extremes of CRL, unless analysed carefully. Altman et al. [17] addressed a similar problem in the estimation of GA using head circumference by restricting the range of measurements included in the regression analyses. As opposed to their HC data, for which the GA range was 12-42 weeks, the INTERGROWTH-21^st^ CRL data span only 5 weeks so using CRL data unaffected by truncation leads to a large loss of data and limited clinical usefulness.

## Conclusion

Although these approaches do not follow standard statistical analysis paradigms for modelling, we have shown empirically that the results of these rather “ad hoc” statistical methods correspond very closely to the “real data” based on the study of Verburg et al. [2], which is a data set similar to CRL data set of the INTERGROWTH-21^st^ project. They are more suitable for large data sets to reduce the effect of sampling variation and ensure reasonable extrapolation. We are thus confident that we can use these approaches to get reliable estimates based on INTERGROWTH-21^st^ CRL data. Although only examined for CRL, these methods may be a solution to other truncation problems involving similar data and their applicability to other settings would need to be evaluated.

### Details of ethics approval

The INTERGROWTH-21^st^ Project was approved by the Oxfordshire Research Ethics Committee ‘C’ (reference:08/H0606/139) and the research ethics committees of the individual participating institutions and corresponding health authorities where the Project was implemented.

## Abbreviations

INTERGROWTH-21st: The International Fetal and Newborn Growth Consortium for the 21^st^ Century; GA: Gestational age; CRL: Crown-rump length; LMP: Last menstrual period; ISUOG: International Society of Ultrasound in Obstetrics and Gynaecology; NICE: National Institute for Health and Care Excellence; FP: Fractional polynomials; SD: Standard deviation.

## Competing interests

The authors declare that they have no competing interests.

## Authors’ contributions

EOO and DGA jointly originated the methodology and concept. EOO performed the statistical analysis and wrote the first draft of the manuscript including figures and tables. All authors contributed to revisions of the manuscript, and read and approved the final version.

## Authors’ information

EOO is a Medical Statistician, AT is Senior Fellow in Fetal Medicine, JV is Professor of Perinatal Medicine and Principal Investigator for the INTERGROWTH-21^st^ Project and DGA is Professor of Medical Statistics.

Eric O. Ohuma^1,2^, Aris T. Papageorghiou^1^, Jose Villar^1^, and Douglas G Altman^2^

^1^Nuffield Department of Obstetrics & Gynaecology and Oxford Maternal & Perinatal Health Institute (OMPHI), Green Templeton College, University of Oxford, Oxford, OX3 9DU, UK: for the International Fetal and Newborn Growth Consortium for the 21st Century (INTERGROWTH-21st Project)

^2^Centre for Statistics in Medicine, University of Oxford, Botnar Research Centre, Windmill Road, Oxford OX3 7LD, UK.

## Pre-publication history

The pre-publication history for this paper can be accessed here:

http://www.biomedcentral.com/1471-2288/13/151/prepub
